# Supplementary material for: Resolving the full spectrum of human genome variation using Linked-Reads
Source: Genome Res. 2019 Apr;29(4):635–45. doi: 10.1101/gr.234443.118 (PMC6442396; doi:10.1101/gr.234443.118)
Supplement: Supplemental Material [file supp_gr.234443.118_Supplemental_longranger-2.2.zip › longranger-2.2.tar.bz2!/lr2.2-public/lib/stan/cmdstan/stan/doc/api/html/check__bounded_8hpp_source.html]

Stan: src/stan/math/error\_handling/check\_bounded.hpp Source File


|  |  |
| --- | --- |
|  | Stan  2.5.0  probability, sampling & optimization |


- Main Page
- Related Pages
- Namespaces
- Classes
- Files

- File List
- File Members

All Classes Namespaces Files Functions Variables Typedefs Enumerations Enumerator Friends Macros Pages

- src
- stan
- math
- error\_handling

check\_bounded.hpp

Go to the documentation of this file.

1 #ifndef STAN\_\_MATH\_\_ERROR\_HANDLING\_\_CHECK\_BOUNDED\_HPP

2 #define STAN\_\_MATH\_\_ERROR\_HANDLING\_\_CHECK\_BOUNDED\_HPP

3

4 #include <stan/math/error\_handling/dom\_err.hpp>

5 #include <stan/math/error\_handling/dom\_err\_vec.hpp>

6 #include <stan/meta/traits.hpp>

7

8 namespace stan {

9  namespace math {

10

11  namespace detail {

12

13  // implemented using structs because there is no partial specialization

14  // for templated functions

15  //

16  // default implementation works for scalar T\_y. T\_low and T\_high can

17  // be either scalar or vector

18  //

19  // throws if y, low, or high is nan

20  template <typename T\_y, typename T\_low, typename T\_high, typename T\_result,

21  bool y\_is\_vec>

22  struct bounded {

23  static bool check(const char\* function,

24  const T\_y& y,

25  const T\_low& low,

26  const T\_high& high,

27  const char\* name,

28  T\_result\* result) {

29  using stan::length;

30  using stan::max\_size;

31  typedef std::pair<typename scalar\_type<T\_low>::type,

32  typename scalar\_type<T\_high>::type> pair\_type;

33

34  VectorView<const T\_low> low\_vec(low);

35  VectorView<const T\_high> high\_vec(high);

36  for (size\_t n = 0; n < max\_size(low, high); n++) {

37  if (!(low\_vec[n] <= y && y <= high\_vec[n]))

38  return dom\_err(function,y,name,

39  " is %1%, but must be between ",

40  pair\_type(low\_vec[n], high\_vec[n]),

41  result);

42  }

43  return true;

44  }

45  };

46

47  template <typename T\_y,

48  typename T\_low,

49  typename T\_high,

50  typename T\_result>

51  struct bounded<T\_y, T\_low, T\_high, T\_result, true> {

52  static bool check(const char\* function,

53  const T\_y& y,

54  const T\_low& low,

55  const T\_high& high,

56  const char\* name,

57  T\_result\* result) {

58  using stan::length;

59  using stan::get;

60  typedef std::pair<typename scalar\_type<T\_low>::type,

61  typename scalar\_type<T\_high>::type> pair\_type;

62

63  VectorView<const T\_low> low\_vec(low);

64  VectorView<const T\_high> high\_vec(high);

65  for (size\_t n = 0; n < length(y); n++) {

66  if (!(low\_vec[n] <= get(y,n) && get(y,n) <= high\_vec[n]))

67  return dom\_err\_vec(n,function,y,name,

68  " is %1%, but must be between ",

69  pair\_type(low\_vec[n], high\_vec[n]),

70  result);

71  }

72  return true;

73  }

74  };

75  }

76

77  // public check\_bounded function

78  template <typename T\_y, typename T\_low, typename T\_high, typename T\_result>

79  inline bool check\_bounded(const char\* function,

80  const T\_y& y,

81  const T\_low& low,

82  const T\_high& high,

83  const char\* name,

84  T\_result\* result) {

85  return detail::bounded<T\_y,T\_low,T\_high,T\_result,

86  is\_vector\_like<T\_y>::value>

87  ::check(function,y,low,high,name,result);

88  }

89

90  }

91 }

92 #endif

dom\_err.hpp

stan::math::check\_bounded

bool check\_bounded(const char \*function, const T\_y &y, const T\_low &low, const T\_high &high, const char \*name, T\_result \*result)

**Definition:** check\_bounded.hpp:79

stan::math::dom\_err

bool dom\_err(const char \*function, const T &y, const char \*name, const char \*error\_msg, const T\_msg error\_msg2, T\_result \*result)

**Definition:** dom\_err.hpp:36

stan::is\_vector\_like

**Definition:** traits.hpp:228

stan::length

size\_t length(const T &)

**Definition:** traits.hpp:159

stan::scalar\_type::type

scalar\_type\_helper< is\_vector< T >::value, T >::type type

**Definition:** traits.hpp:139

stan::math::dom\_err\_vec

bool dom\_err\_vec(const size\_t i, const char \*function, const T &y, const char \*name, const char \*error\_msg, const T\_msg error\_msg2, T\_result \*result)

**Definition:** dom\_err\_vec.hpp:33

traits.hpp

stan::max\_size

size\_t max\_size(const T1 &x1, const T2 &x2)

**Definition:** traits.hpp:191

stan::math::detail::bounded

**Definition:** check\_bounded.hpp:22

stan::math::detail::bounded< T\_y, T\_low, T\_high, T\_result, true >::check

static bool check(const char \*function, const T\_y &y, const T\_low &low, const T\_high &high, const char \*name, T\_result \*result)

**Definition:** check\_bounded.hpp:52

dom\_err\_vec.hpp

stan::get

T get(const T &x, size\_t n)

**Definition:** traits.hpp:143

stan::math::detail::bounded::check

static bool check(const char \*function, const T\_y &y, const T\_low &low, const T\_high &high, const char \*name, T\_result \*result)

**Definition:** check\_bounded.hpp:23

stan::VectorView

VectorView is a template metaprogram that takes its argument and allows it to be used like a vector...

**Definition:** traits.hpp:275


---

[ Stan Home Page ]

*© 2011–2014,
Stan Development Team.*
